# Histological Analyses Demonstrate the Temporary Contribution of Yolk Sac, Liver, and Bone Marrow to Hematopoiesis during Chicken Development

**DOI:** 10.1371/journal.pone.0090975

**Published:** 2014-03-12

**Authors:** Priscila Tavares Guedes, Barbara Cristina Euzébio Pereira Dias de Oliveira, Pedro Paulo de Abreu Manso, Luzia Fátima Gonçalves Caputo, Gerson Cotta-Pereira, Marcelo Pelajo-Machado

**Affiliations:** 1 Laboratory of Pathology, Oswaldo Cruz Institute/Fiocruz, Rio de Janeiro, Brazil; 2 Laboratory of Immunochemistry and Histochemistry, Santa Casa da Misericórdia do Rio de Janeiro, Rio de Janeiro, Brazil; Peking University, China

## Abstract

The use of avian animal models has contributed to the understanding of many aspects of the ontogeny of the hematopoietic system in vertebrates. However, specific events that occur in the model itself are still unclear. There is a lack of consensus, among previous studies, about which is the intermediate site responsible for expansion and differentiation of hematopoietic cells, and the liver's contribution to the development of this system. Here we aimed to evaluate the presence of hematopoiesis in the yolk sac and liver in chickens, from the stages of intra-aortic clusters in the aorta-genital ridges-mesonephros (AGM) region until hatching, and how it relates to the establishment of the bone marrow. *Gallus gallus domesticus* L. embryos and their respective yolk sacs at embryonic day 3 (E3) and up to E21 were collected and processed according to standard histological techniques for paraffin embedding. The slides were stained with hematoxylin-eosin, Lennert's Giemsa, and Sirius Red at pH 10.2, and investigated by light microscopy. This study demonstrated that the yolk sac was a unique hematopoietic site between E4 and E12. Hematopoiesis occurred in the yolk sac and bone marrow between E13 and E20. The liver showed granulocytic differentiation in the connective tissue of portal spaces at E15 and onwards. The yolk sac showed expansion of erythrocytic and granulocytic lineages from E6 to E19, and E7 to E20, respectively. The results suggest that the yolk sac is the major intermediate erythropoietic and granulopoietic site where expansion and differentiation occur during chicken development. The hepatic hematopoiesis is restricted to the portal spaces and represented by the granulocytic lineage.

## Introduction

Hematopoiesis is the process of proliferation, self-renewal, and differentiation of hematopoietic stem cells (HSCs) resulting in blood cell formation during both embryonic and adult stages [1]–[5]. In the last decades, progress in understanding the development of HSCs in vertebrates has been made through studies using different animal models, including avian embryo models [6].

Avian animal models are among the oldest vertebrate models used in developmental biology and have been essential to clarify the origin of definitive HSCs. The study of chicken–quail and chicken–chicken chimeras, built before the fusion of intra- and extra-embryonic vascular circuits, showed that definitive HSCs do not arise from the yolk sac but in the embryo itself [7], [8]. Subsequently, evaluations of the grafting of quail embryo aorta into the chicken embryo dorsal mesentery [9] and *in vitro* clonal assays [10], [11] established evidence that the chicken dorsal aortic region is the source of intra-embryonic HSCs. This hematopoietic activity in analogous area in chickens was previously verified in amphibians [12]. Further investigations in mouse embryos by CFU-S assay showed that the AGM (aorta-genital ridges-mesonephros) region is an enriched source of hematopoietic activity [13]. Subsequent experiments proving the intra-embryonic origin of definitive HSCs in the para-aortic splanchnopleura/AGM region in murine model showed that the steps involved in the avian hematopoietic development basically apply to higher vertebrates [14]. Moreover, a similar role for the AGM region was identified in allantois of chicken embryos [15], [16]. Because the mouse placenta has an allantois component, the findings obtained in chicken embryos led to the study of hematopoietic activity in mouse placenta, which demonstrated that this organ is a major hematopoietic organ during mouse development [17].

The use of avian animal models has contributed to the understanding of hematopoietic events in mammals; however, some specific aspects of the avian hematopoietic system ontogeny, such as the site where expansion and differentiation of hematopoietic cells occurs, remain unclear [6]. This role is attributed to the fetal liver in mammals [18]–[20] and seems to be assumed by the para-aortic foci in chickens [6] However, erythropoiesis and granulopoiesis was seen in a short developmental period in this site [21]. Conversely, the hematopoietic process has been described in the yolk sac during a significant part of the embryonic life [22]–[25]. The chicken fetal liver was initially considered a non-hematopoietic organ [22], however displaying intra- and/or extra-vascular hematopoiesis [23], [26]–[29]. Thus, correlations between the chicken fetal liver and other hematopoietic structures and the understanding of the hematopoietic process in more detail demand further investigation. An integrated morphological study is important to better understand the temporal and spatial distribution of hematopoietic sites during avian development.

In this study, we used histological techniques to evaluate the presence of hematopoiesis in the yolk sac and liver of chickens during development, from the stages of intra-aortic clusters in the AGM region until hatching, and how it relates to the establishment of the bone marrow. We found that there is a topographic and temporal relationship of the hematopoietic activity among the yolk sac, fetal liver, and bone marrow during chicken development. We also showed that the yolk sac is the major expansion and differentiation site of erythrocytic and granulocytic lineages whereas the liver's contribution to hematopoietic activity is limited and restricted to the granulocytic population.

## Materials and Methods

### Ethics Statement

All procedures with chicken embryos were performed according to the ethical recommendation of the Ethics Committee from the Fundação Oswaldo Cruz (Oswaldo Cruz Foundation).

### Animals

Fertilized White Leghorn chicken eggs (*Gallus gallus domesticus* L.) were purchased from commercial sources (Tolomei Farms, Rio de Janeiro, Brazil) and incubated at 37.5°C and 55% relative air humidity. The embryos and their respective yolk sacs were collected daily between embryonic days 3 (E3) and E21 (from the beginning of intra-aortic clusters formation in the AGM region stages until hatching). The embryos were staged according to morphological parameters proposed by Hamburger and Hamilton (HH) [30].

### Sampling and histological processing

Yolk sacs were separated from the embryos using scissors. The yolk was removed and the yolk sac stretched and transferred to a glass dish. Embryo and yolk sac were washed in phosphate-buffered saline (PBS) at pH 7.2 and fixed for 48 hours in Carson's Millonig formalin at room temperature [31]. The embryos were staged after 24 hours of fixing, and yolk sacs and embryos were subsequently cleaved. Embryos' wings and legs were separated from the body at E5 and onwards. The trunks of E3 to E18 embryos were transversely sectioned into subsequent samples of about 3 mm; organs from E19 and onward embryos were cleaved after being dissected. Yolk sacs were cleaved in regions defined by quadrants. Samples were processed in a Shandon Citadel 2000 tissue processor (Thermo, USA) according to standard histological techniques for paraffin embedding. Five-micrometer-thick paraffin serial sections were obtained in a rotary microtome (Microm HM-325). These sections were de-waxed, hydrated, and washed in distilled water to prepare for staining with hematoxylin-eosin [32], Lennert's Giemsa [33], and Sirius Red at pH 10.2 staining [34], [35]. Slides with sections from yolk sacs, AGM region, livers, and long bones were analyzed in an Axioskop microscope (Carl Zeiss, Germany). The images were acquired with an AxioCam MRc5 color camera (Carl Zeiss, Germany).

## Results

The morphological criteria used to determine hematopoietic foci were based on the presence of clusters constituted by blood cells at various stages of maturation and mitosis profiles.

### Chicken yolk sac hematopoiesis: expansion of erythropoiesis and granulopoiesis

**Figure 1:**
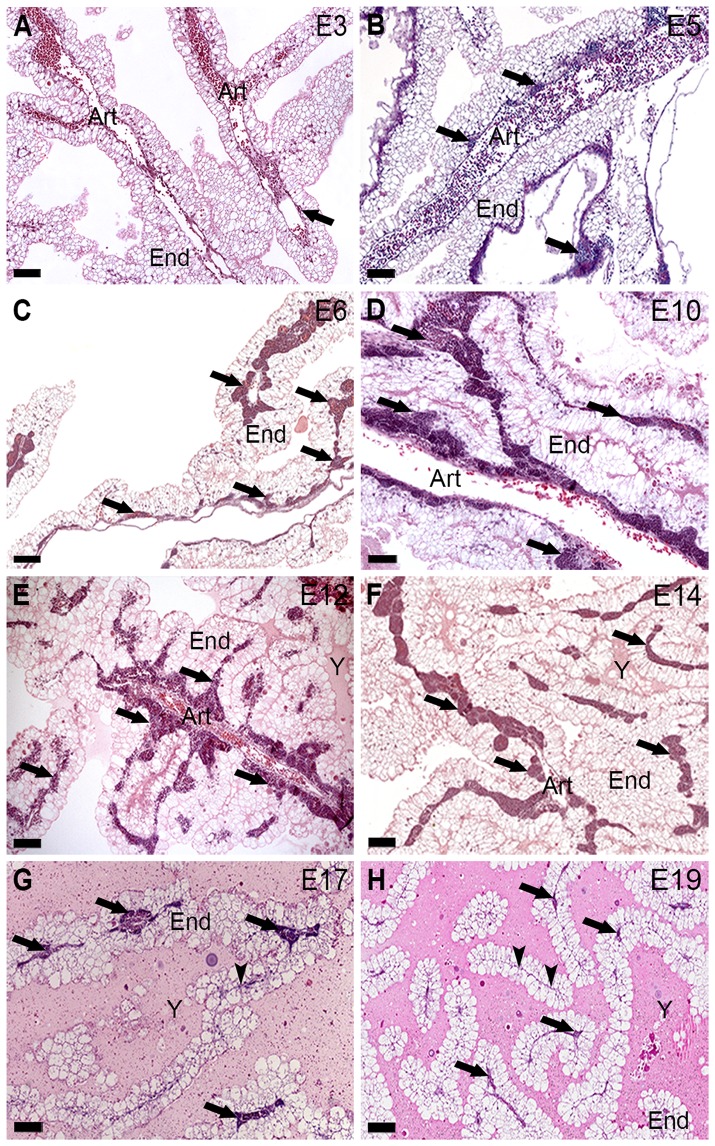
(A–H) Panoramic view of chicken yolk sacs between E3 and E19. The embryonic day (E) is indicated in the upper right corner in each picture. Histological sections of yolk sacs show endoderm (End), vessels (Art), and areas of hematopoietic foci (arrows). (**A**–**E**) The areas occupied by these foci are shown gradually increasing in the photomicrographs between E3 and E12, and (**F**–**H**) decreasing in onwards stages. (**G, H**) Atrophic vessels (arrowheads). (**F**–**H**) Yolk (Y). Hematoxylin-eosin. Bars 100 µm.

**Figure 2:**
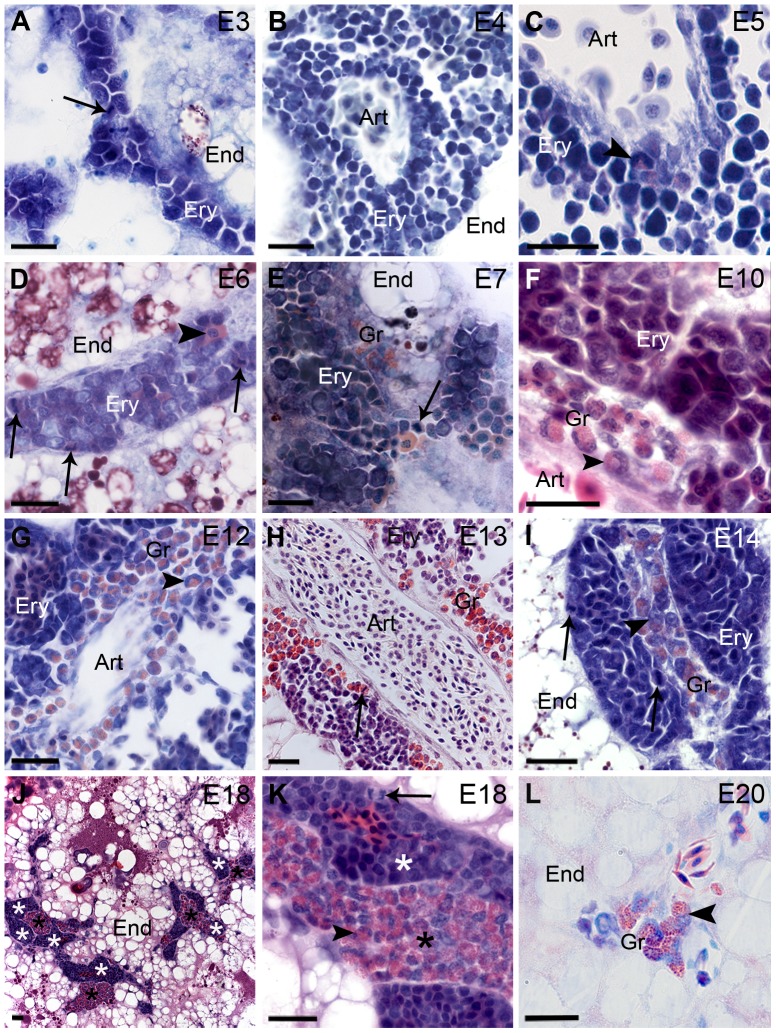
(A–K) Erythropoiesis (Ery) and (E–L) granulopoiesis (Gr) in chicken yolk sac between E3 and E20. The embryonic day (E) is indicated in the upper right corner in each picture. Thin arrows show mitosis in erythrocytes (**A**, **D**, **E**, **I**, **K**) and in a granulocyte (**H**). (**A**) Predominance of basophilic cells with a slight acidophily. (**B**, **C**) Numerous pro-erythroblasts and basophilic erythroblasts (Ery) between artery (Art) and endoderm (End). (**C**) Note a cell band leukocyte (arrowhead). (**D**) Erythrocytic (Ery) focus showing mature erythrocyte (arrowhead). (**F**, **G**) Cell band leukocyte (arrowhead). (**H**) Eosinophil granules into granulocytic cells at different stages of maturation show the cytoplasm of these cells in red-orange color. (**I**) Promyelocyte (arrowhead). (**J**, **K**) Foci of erythrocytic (white asterisks) and granulocytic (black asterisks) differentiation are present in equivalent numbers at this stage. (**K**) Mature leukocyte (arrowhead). (**L**) Myelocyte (arrowhead). (**E**–**K**) Note that granulocytic and erythrocytic lineages do not mix. End, endoderm; Art, artery. (**A**–**E**, **G**, **I**, **L**) Lennert's Giemsa, (**F**, **J**, **K**) Hematoxylin-eosin, and (**H**) Sirius Red stains at pH 10.2. Bars 20 µm.

The panoramic view of the histological sections from yolk sacs, taken during chicken development, showed endodermic cells compounding the endoderm, vessels, and areas of hematopoiesis (Fig. 1). Erythrocytic and granulocytic populations constituted these areas (Fig. 2). Hematopoiesis was seen in the yolk sac from E3 (HH20, 21) to E20 (HH45) (Figs. 1, 2).

Hematopoietic foci were rare at E3 (HH20, 21; when intra-aortic clusters were noted in the AGM region) (Fig. 1A), however, gradually enhanced at E4 (HH25) and onwards (Fig. 1B–E). Starting at E14 (HH40), these areas gradually decreased (Fig. 1F–H) showing rare foci at E19 and E20 (HH45) (Fig. 1H). The vessels undergo atrophy in the last stages (Fig. 1G, H).

Foci represented the erythrocytic population and were constituted by basophilic cells with slight acidophily at E3 (HH20, 21) (Fig. 2A). A great number of pro-erythroblasts and basophilic erythroblasts were located between the endoderm and vitelline arteries in yolk sacs at E4 (HH24, 25) (Fig. 2B) and E5 (HH27, 28) (Fig. 2C). Granulopoietic foci were not commonly seen at E4 and E5; however, rare mature leukocytes or ones at different stages were verified (Fig. 2C). The complete erythrocytic differentiation cascade was observed starting at E6 (HH29, 30) and included mature erythrocytes (Fig. 2D). Mitosis in erythrocytic cells was observed and persisted in later stages (Fig. 2A, D, E, I, K). An erythrocytic population was observed in yolk sacs until E19 (HH45). Granulocytic foci were observed in chicken yolk sacs formed by promyelocytes, myelocytes, cell bands, and mature leukocytes (Fig. 2E–I, K, L) full of eosinophil granules from E7 (HH32) to E20 (HH45) (Fig. 2E–L). Mitosis was detected in granulocytic cells persisting in later stages (Fig. 2H). Erythropoietic and granulopoietic foci were observed near but separated from each other (Fig. 2E–K).

While the erythropoiesis progressively started to decrease at E14/15 (HH40, 41), the granulopoiesis remained stable during the second half of the developmental period until E18 (HH44) (Fig. 2 J, K), however, reducing in later stages (Fig. 2L).

### Liver characteristics and hematopoiesis: granulopoiesis around portal vessels at later stages in the developmental period

**Figure 3:**
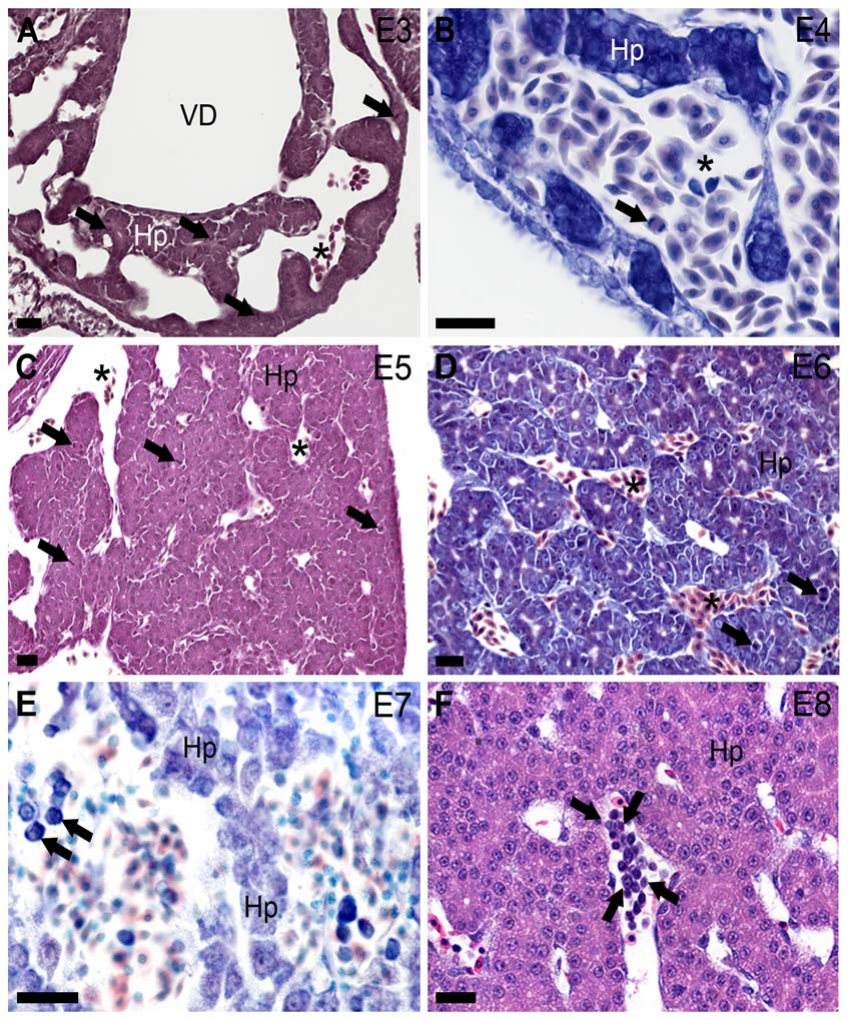
Chicken liver development without hematopoietic activity from E3 to E8. The embryonic day (E) is indicated in the upper right corner in each picture. (**A**, **C**, **D**) Numerous mitosis (arrows) are seen in hepatoblasts (Hp). (**B**) Mitosis in circulating erythrocyte (arrow). (**E**) Immature hematopoietic circulating cells (arrows) in sinusoidal capillaries (vessels located between hepatoblast cords, Hp). Note the large and irregular lumen of the sinusoidal capillaries. (**F**) Foci of immature erythropoietic cells in circulation (limited by arrows). Hp, hepatoblasts; VD, venous duct; sinusoidal capillaries (asterisks). (**A**, **C**, **F**) Hematoxylin-eosin and (**B**, **D**, **E**) Lennert's Giemsa stains. Bars 20 µm.

**Figure 4:**
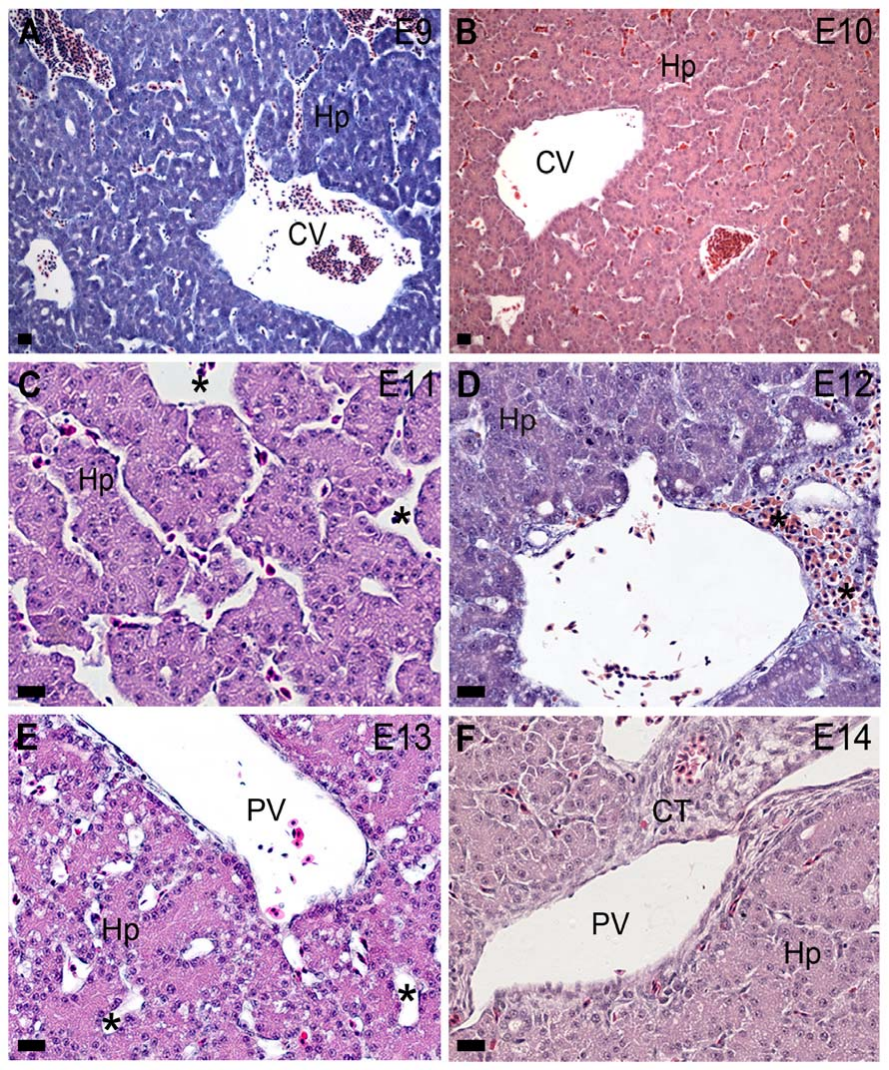
Chicken liver development without hematopoietic activity from E9 to E 14. The embryonic day (E) is indicated in the upper right corner in each picture. Hp, hepatoblasts; CV, central vein; sinusoidal capillaries (asterisks); PV, portal vein; CT, connective tissue. (**A**) Lennert's Giemsa and (**B**–**F**) Hematoxylin-eosin stains. Bars 20 µm.

**Figure 5:**
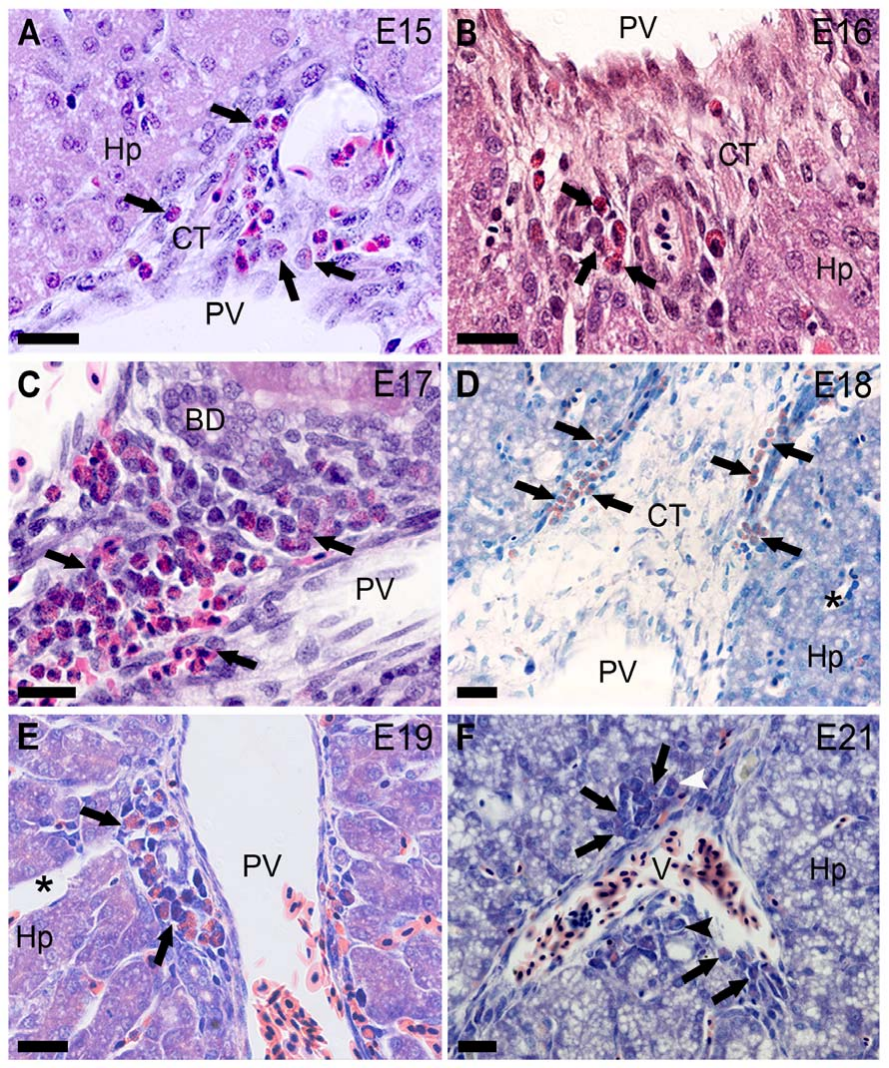
Liver development with hematopoietic activity (E15 to E19 and E21). The embryonic day (E) is indicated in the upper right corner in each picture. Granulopoiesis (limited by arrows) is demonstrated in connective tissue (CT) in the liver portal spaces (PV, portal vein; BD, biliary duct; V, vessel). (**F**) Promyelocyte (black arrowhead). Myelocyte (white arrowhead). (**A**–**C**) Hematoxylin-eosin and (**D**–**F**) Lennert's Giemsa stains. Bars 20 µm.

The temporal analysis of the liver in chickens, between the AGM stages and hatching, showed that the proliferative aspect and morphological changes of hepatocytes are intense, especially in the early days (Fig. 3A–D), when compared to the later stages (Figs. 3E, F; 4A–F and 5A–F).

Hematopoiesis was not verified in the liver until E14 (HH40) (Figs. 3A–F and 4A–F) despite the presence of immature hematopoietic circulating cells (Fig. 3E, F). Some immunoblastoid phenotype cells were identified at around E7–8 (HH32–34) (Fig. 3E). Hematopoiesis was verified in the liver at E15 (HH41) and onwards. Granulocytic was the main lineage of cells observed in the liver in connective tissues of hepatic portal spaces, however, not among hepatocytes (Fig. 5A–F). Nevertheless, not all connective tissue areas in portal spaces were occupied by granulopoietic foci (Fig. 5B, D).

### Hematopoietic activity in the marrow of long bones during chicken developmental stages

**Figure 6:**
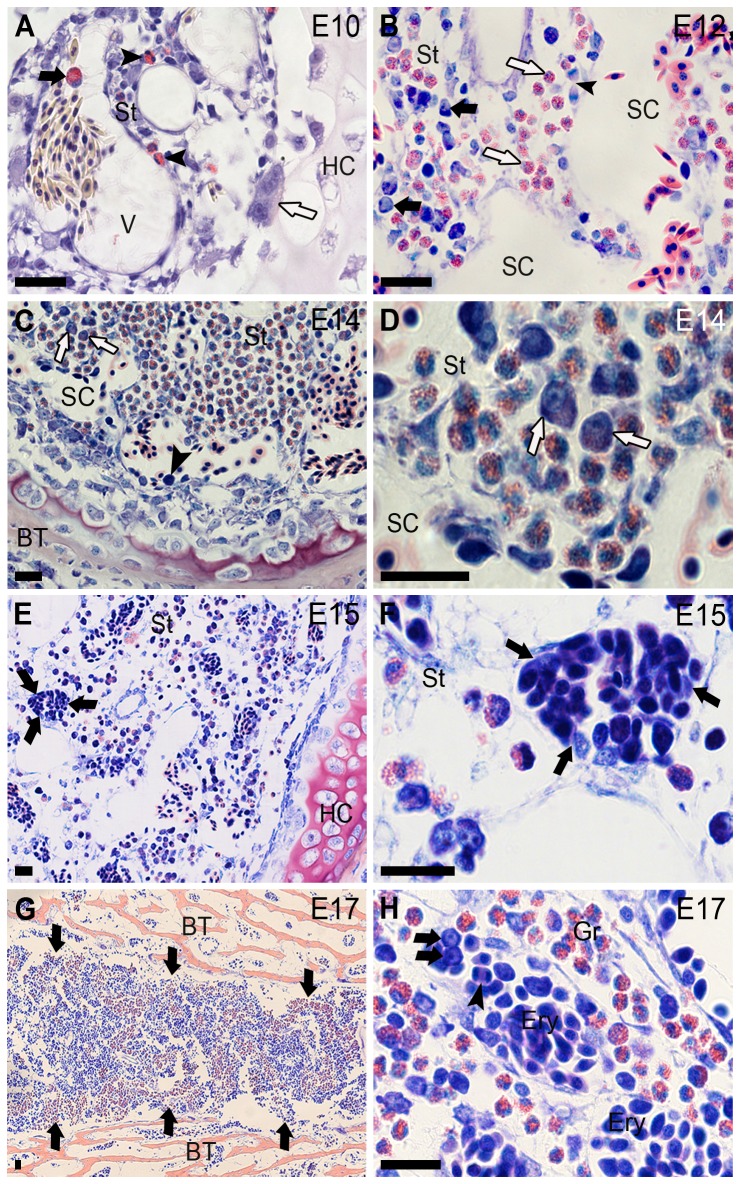
Establishment of hematopoiesis in the diaphysis of long bones during chickens' developmental period. The embryonic day (E) is indicated in the upper right corner in each picture. (**A**) Leukocytes with eosinophil granules (arrowheads) are shown in the marrow stroma (St) in the beginning of the bone marrow formation. Metamyelocyte (black arrow) into vessel (V). Osteoclast (white arrow). (**B**) Granulocytes (white arrows) and basophilic cells with eccentric nucleus and prominent nucleolus (black arrows) are shown in the marrow stroma. Mitosis in erythrocyte (arrowhead). (**C**) Basophilic cell (arrowhead). Granulocytes at different stages of maturation in the stroma including promyelocytes (arrows). (**D**) These cells are shown in high magnification (arrows). (**E**) Granulocytes in the stroma (St) and erythropoiesis (limited by arrows) in sinusoidal capillaries, shown (**F**) in high magnification. (**G**) Panoramic view of a transversal section of diaphysis. The bone marrow (limited by arrows) shows areas of erythropoiesis (blue color) and granulopoiesis (red color). (**H**) Erythropoiesis (Ery) in sinusoidal capillaries showing immature hematopoietic cells (arrows) and mitosis in erythrocyte (arrowhead). Granulopoiesis (Gr) in the marrow stroma. HC, hypertrophic cartilage; SC, sinusoidal capillary; BT, bone tissue. (**A**) Sirius Red at pH 10.2, and (**B**–**H**) Lennert's Giemsa stain. Bars 20 µm.

Rudiments of chicken long bones at E8 (HH34) and E9 (HH35) were composed of cartilaginous tissue with hypertrophic chondrocytes in the center of the diaphysis. In this area, at E10 (36HH), the bone marrow cavity was seen with osteoblasts closely surrounded by cartilage islets and osteoclasts (Fig. 6A). In this phase, granulocytes with specific granules like myelocytes, metamyelocytes, and mature leukocytes were noted in the bone marrow stroma and circulating into bone marrow vessels (Fig. 6A). Between E11 and E13 (37–39HH), the bone marrow stroma was accompanied by granulocytes at the same differentiation stages observed at E10, however, basophilic cells were observed among them (Fig. 6B). Granulopoiesis was observed associated to the bone marrow stroma at E14 (40HH) (Fig. 6C), characterized by the presence of promyelocytes (Fig. 6D). Some erythropoietic foci were seen from E13 (39HH) in bone marrow vessels; however, these foci were frequently observed at E14 (40HH) and E15 (41HH) (Fig. 6E, F). Granulopoiesis and erythropoiesis gradually rose after E14 and E15 in the parenchyma and bone marrow vessels, respectively (Fig. 6G, H).

## Discussion

This study used histological techniques to evaluate the presence of hematopoiesis in chickens' yolk sac and liver, during their development, from the stages of intra-aortic clusters in the AGM region until hatching, and how it relates to the establishment of the bone marrow.

**Figure 7:**
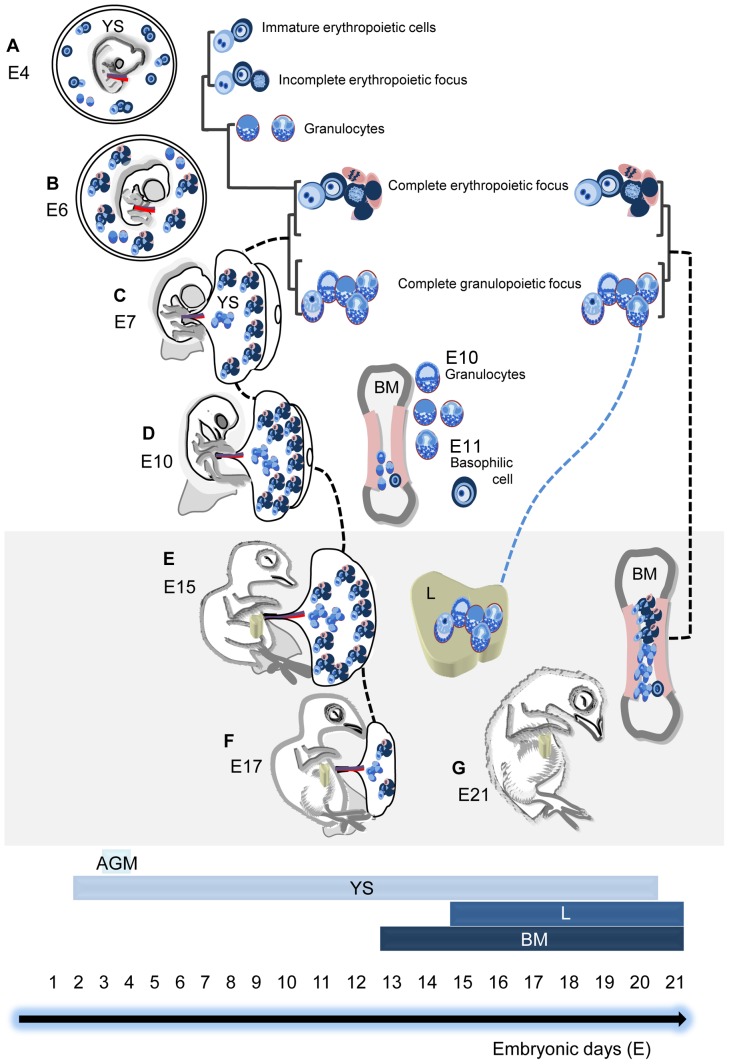
Scheme of hematopoiesis in the yolk sac, liver, and bone marrow during chicken development. Bars indicate the temporal distribution of this activity in the AGM region, yolk sac (YS), liver (L), and bone marrow (BM). Black dotted lines indicate the presence of both erythropoiesis and granulopoiesis in the YS (**C**–**F**) and BM (**E**–**G**). The blue dotted line is to draw attention to the granulopoiesis in the L. (**A**) At E4, immature erythropoietic cells, incomplete erythropoietic foci, and rare granulocytes are distributed in the YS. (**B**) From E6, the YS shows complete erythropoietic foci (all maturation stages are seen) and some granulocytes. (**C**) From E7, complete erythropoietic and granulopoietic foci are distributed in the YS. (**D**) At E10, erythropoietic and granulopoietic foci are seen in the YS. Granulocytes at different stages of maturation are noted in the BM. At E11, basophilic cells are also seen in the BM. (**E**) At E15, both erythropoietic and granulopoietic foci are frequently observed in the YS and BM. In this phase, granulopoiesis begins in the L around the portal vessels. (**F**) At E17, hematopoiesis is reduced in the YS. Granulopoiesis persists in the L portal spaces, and both erythropoietic and granulopoietic activities are noted in the BM. (**G**) At E21, granulopoietic foci are seen in L connective tissues, both erythrocytic and granulocytic activities are observed in the BM, and the YS is no longer a hematopoietic site.

We observed that the yolk sac is the major hematopoietic site during the development of chickens and that some expansion and differentiation phenomena, at least for the erythrocytic and granulocytic cell lineages, occurred. In this animal model, the liver contributes to granulopoiesis, which is restricted to the perivascular connective tissue at E15 and onwards. The hematopoietic colonization of the bone marrow starts at E13 and intensifies at E14 and E15 together with a progressive reduction in hematopoietic activity in the yolk sac. Figure 7 and a supplementary video (Video S1) show a schematic view of the data.

At the beginning of the twentieth century, Dantschakoff [22] presented the yolk sac as the major hematopoietic structure during chicken development and proposed using chicken yolk sacs for morphological characterization of the erythrocytic and granulocytic cascades. In this author's analysis, a great number of large lymphocytes, at different maturation stages, were seen from the fourth day in the developmental period. At that time, lymphocytes represented HSCs, which were characterized as basophilic cells with eccentric nucleus, one or two nucleoli, and discrete cytoplasmic acidophily depending on the degree of differentiation. In addition, according to Dantschakoff [22], when these cells settled into yolk sac vessels, they differentiated in mature erythrocytes; however, when they were in the extravascular compartment, they acquired eosinophilic granules gradually changing nucleus conformation and originating myelocytes and mature leukocytes. This topographic profile was also shown in bone marrow in embryonic and adult chickens and other adult avian species by Dantschakoff [36]. Although the current nomenclature does not completely correspond to that described by Dantschakoff [22], there have been a few further reports about granulopoiesis and the morphological description of hematopoietic lineages of chicken yolk sacs, especially in the second half of the developmental period.

In the present study, erythropoietic cells distributed in chicken yolk sacs at E4 and E5 consist mainly of pro-erythroblasts and basophilic erythroblasts, while the entire erythrocytic cascade was observed from E6 to E19. Considering the temporality and similarity between the morphological description of Dantschakoff [22] and the cells analyzed in this study, we suggest that the Dantschakoff's lymphocytes [22] represent pro-erythroblasts, basophilic, and polychromatophilic erythroblasts, which does not rule out the possibility that HSCs were present between them. Erythrocytic foci in chicken yolk sacs after the AGM stages have been confirmed as belonging to definitive erythropoiesis by either of the following analyses: beta A protein transcripts between E4 and E15 [37], or benzidine staining from E5 to E19 [25]. In the present study, the erythropoietic foci observed in yolk sacs from E6 and onwards consisted of cells at different stages of maturation, from pro-erythroblasts to mature erythrocytes. The location of erythropoietic foci observed in our results is comparable to the images of definitive erythropoiesis foci provided by Nagai and Sheng [37] and in agreement with the data reported by Niimi et al [25] indicating observation of definitive erythropoietic foci up to E19. Thus, our data combined with the data reported in the literature, suggest that the erythropoietic cells are committed to definitive lineages at different stages of maturation in the yolk sac itself.

Dieterlen-Lièvre [7] suggested that blood cells originated from the embryo may be found in the yolk sac vessels after they reach the blood circulation. In our study, a change from rare erythropoietic foci in chicken yolk sacs during AGM intra-aortic clusters stages (E3) to a great number of immature hematopoietic cells in subsequent stages (E4, E5) was observed. Our data suggest that at least some of the intra-embryonic HSCs originated from the intra-aortic clusters promptly seed the chicken yolk sac where they expand. Findings reported by Dieterlen-Lièvre et al [38] showed that red blood cells of intra-embryonic origin are in the blood circulation in chick yolk sac chimeras from E6. These cells increased in number and showed to be the major blood component of the chimeras at E13. In our results, erythropoietic foci enhanced in the middle development period in the chicken yolk sacs and they revealed mature erythrocytes from E6. Combining the findings reported here, it is reasonable to speculate that the intra-embryonic HSCs from dorsal aorta are the precursors of the hematopoietic cell lineages undergoing expansion and differentiation processes in the chicken yolk sac and, consequently, they provide the mature erythrocytes in the blood circulation.

The detection of the ability of the yolk sac to expand the erythropoietic and granulopoietic lineages is noteworthy. We showed that granulopoietic foci are composed by promyelocytes, which subsequent stages mature into leukocytes. The concentration of these cells is rare in yolk sacs after the AGM proliferative stages but becomes frequent after E6. In addition, the abundance of these foci was observed in the second half of the chicken developmental period although their concentration did not exceed the concentration of erythropoietic foci until E18. At E18, the extension occupied by erythropoiesis and granulopoiesis foci was similar in yolk sacs. Therefore, our results show the role of the yolk sac as a differentiation and expansion site for both erythropoietic and granulopoietic lineages. These lineages occupy different niches and remain separated.

The chicken fetal liver is not considered a relevant hematopoietic organ, as is the fetal liver in mammals [23], [26], [28], [29], [39]; however, intra- or extra-vascular hematopoiesis occurs in this organ [23], [26], [27], [29]. Haff [26] considered two important moments in hematopoiesis during the development of the chicken liver. The first corresponds to erythropoiesis within liver capillaries between E7 and E9. The second corresponds to granulopoiesis in the connective tissues. This author reports that granulopoiesis begins at E11, reaches its peak between E14 and E15, and is gradually reduced, ceasing during hatching. However, Kingsbury et al [27], who observed the development of chicken livers from 33 hours of development, verified the presence of eosinophilic cells, both intra- and extravascular, initially at E14. These cells increased in number at E15 and were observed forming extravascular clusters between E17 and E18.

Our study shows that no hematopoiesis occurs in the hepatic parenchyma during the entire embryonic development in *Gallus gallus domesticus* L. We observed circulating erythropoietic cells at different maturation stages in sinusoidal capillaries on the same days described by Haff [26]. Even considering that erythropoiesis in birds is intra-vascular, immature erythrocytic cells do not form foci as observed in the yolk sac in this study. Conversely, granulocytic cells were often seen, sometimes resembling granulopoiesis, around the connective tissue in portal spaces. Thus, our data agree with the findings that described granulocytes in connective tissues [26], [27], [29], however, closer to the stages when bone marrow starts its granulopoietic activity at hatching (E14 to E21). In addition, our findings support the fact that some of these cells are in the process of differentiation and that not all of the connective tissue extension is occupied by them. The accumulation of these granulocytic cells in the chicken fetal liver could be explained by their possible migration out of the yolk sac and into the bone marrow, where a microenvironment is suitable for hematopoiesis in the connective tissue.

Vasse and Beaupain [40] and Ayres-Silva et al [41] reported the same late colonization of granulocytic cells in fetal liver connective tissue in the turtle *Emys orbicularis* L. and in mice, respectively, suggesting a phylogenetic conservation of this event between these vertebrates and chickens. However, it is unlikely that granulocyte progenitors in mice that come from the yolk sac arrive late in fetal liver because the hematopoietic activity in the yolk sac ceases at 11.5 days post-coitum [41]. Moreover, granulopoiesis in the liver does not seem to be an exclusive event of fetal development. Jordan [42] showed the presence of granulopoietic foci in connective tissue in portal spaces and subcapsular interstitial tissue using histological analysis, in 1- and 3-month-old chickens and pigeons. Vasse and Beaupain [40] showed the persistence of cortical granulopoiesis in turtles that were at least 2 years old.

Sheng [43] suggested that the chicken bone marrow becomes the major erythropoietic organ, sometime between E12 and E15, although the yolk sac remains to contribute to erythropoiesis close to hatching. In the present study, we observed that some erythropoietic foci could be seen at E13 in the marrow of long bones, but they were frequent at E14 and E15, and progressively enhanced onwards. Based on our results, it is likely that bone marrow in *Gallus gallus domesticus* L. is the major erythropoietic organ from E14 and E15 and onwards related to a gradual erythropoiesis reduction in the yolk sac in this period of incubation. However, our results do not estimate the first moment when the bone marrow becomes the main hematopoietic organ because this would require a systematic study of all sites of ossification, from the first moments of this phenomenon to the establishment of hematopoietic activity in the bone marrow of all bones in *Gallus gallus domesticus* L.

Unlike in erythropoiesis, our results did not show a granulopoiesis relationship between the yolk sac and bone marrow establishment. In agreement with the results reported by Dantschakoff [36], we observed eosinophilic granulocytes at different stages of maturation in the bone marrow at E12, and the appearance of granulopoiesis at E14, which increased in later stages of development in this organ. However, in the second half of the chickens' developmental period, the granulopoietic activity remained apparently constant in the yolk sac, even when the bone marrow had already shown hematopoietic establishment.

The present study identified that the fetal liver is not the main hematopoietic expansion site in chickens based on an integrated analysis of the liver, yolk sac, and bone marrow. On the contrary, we observed hematopoiesis in the yolk sac during almost the entire developmental period of chickens. While this structure is present in reptiles and birds until hatching, it involutes in mammals [23], [40], [44], [45]. In reptilian species, the yolk sac is also the main erythropoietic or hematopoietic structure [40], [46]. In mammals, hematopoietic cells from the yolk sac and AGM region migrate to the fetal liver [13], [20], [47], [48], which is considered the major intermediate expansion hematopoietic compartment in these animals. According to Wong and Cavey [29], the crucial elements of the hematopoietic environment in the mammalian liver may not have the same importance in birds, due to intravascular erythropoiesis in these animals.

We are aware of the histological technique limitation used to the present study. However, this method made clear the integrated and temporary contribution of the hematopoietic sites to expansion and differentiation of the erythrocytic and granulocytic lineages during chicken development. Moreover, it indicated the presence of immature hematopoietic cells. They were diagnosed based on their morphological characteristics and their location among erythrocytic/granulocytic cells or inside vessels [21]. Further investigation, applying immunohistochemistry and/or in situ hybridization, would be important to deep our results, since these methods could distinguish the different hematopoietic lineages and also more primitive HSCs.

In conclusion, our results showed that the yolk sac is the major expansion and differentiation site of granulopoiesis and erythropoiesis during the chickens' developmental period, even when the bone marrow acquires functionality and granulocytes are observed in the liver portal spaces. Moreover, it seems that the yolk sac involution in mammals favors other phylogenetic adaptations such as the hepatic microenvironment for hematopoietic expansion.

## Supporting Information

Video S1 **The video S1 is related to** **Figure 7** **and shows a schematic view of the temporal contribution of the yolk sac, liver, and bone marrow to erythropoiesis and granulopoiesis in each day of the chicken development, from the AGM stages (E3) until hatching day (E21).** Total time: 3:40 minutes (WMV).
(WMV)
